# Circadian rhythm of cardiac electrophysiology, arrhythmogenesis, and the underlying mechanisms

**DOI:** 10.1016/j.hrthm.2018.08.026

**Published:** 2019-02

**Authors:** Nicholas Black, Alicia D’Souza, Yanwen Wang, Hugh Piggins, Halina Dobrzynski, Gwilym Morris, Mark R. Boyett

**Affiliations:** ∗ Division of Cardiovascular Sciences, University of Manchester, Manchester, United Kingdom; † Division of Diabetes, Endocrinology & Gastroenterology, University of Manchester, Manchester, United Kingdom

**Keywords:** Arrhythmia, Autonomic nervous system, Cardiac conduction system, Cardiac pacemaking, Circadian rhythm, Ion channel remodeling

## Abstract

Cardiac arrhythmias are a leading cause of cardiovascular death. It has long been accepted that life-threatening cardiac arrhythmias (ventricular tachycardia, ventricular fibrillation, and sudden cardiac death) are more likely to occur in the morning after waking. It is perhaps less well recognized that there is a circadian rhythm in cardiac pacemaking and other electrophysiological properties of the heart. In addition, there is a circadian rhythm in other arrhythmias, for example, bradyarrhythmias and supraventricular arrhythmias. Two mechanisms may underlie this finding: (1) a central circadian clock in the suprachiasmatic nucleus in the hypothalamus may directly affect the electrophysiology of the heart and arrhythmogenesis via various neurohumoral factors, particularly the autonomic nervous system; or (2) a local circadian clock in the heart itself (albeit under the control of the central clock) may drive a circadian rhythm in the expression of ion channels in the heart, which in turn varies arrhythmic substrate. This review summarizes the current understanding of the circadian rhythm in cardiac electrophysiology, arrhythmogenesis, and the underlying molecular mechanisms.

## Introduction

A circadian rhythm is an oscillation of a physiological process over a 24-hour period. Many cardiovascular variables, including heart rate, heart rate variability (HRV), electrocardiogram (ECG) waveforms, and blood pressure, demonstrate a robust circadian rhythm.^1^ Strictly speaking, an oscillation is considered circadian only if it persists over 24 hours of darkness. In many cases, this has not been tested for cardiovascular variables, but nevertheless the oscillations are here described as circadian because the term is in common usage. Many cardiovascular diseases vary in prevalence by time of day, including myocardial infarction, supraventricular/ventricular arrhythmias, and sudden cardiac death (SCD).^1^ This review aims to bring up to date our knowledge of the circadian rhythm in cardiac electrophysiology and arrhythmogenesis and, most importantly, to discuss the underlying mechanisms. Data shown are from the human or rodents; unlike humans, rodents are nocturnal and are active at night and sleep during the day. The text here corresponds to the human unless specifically stated otherwise.

### Circadian clocks

**Figure 1:**
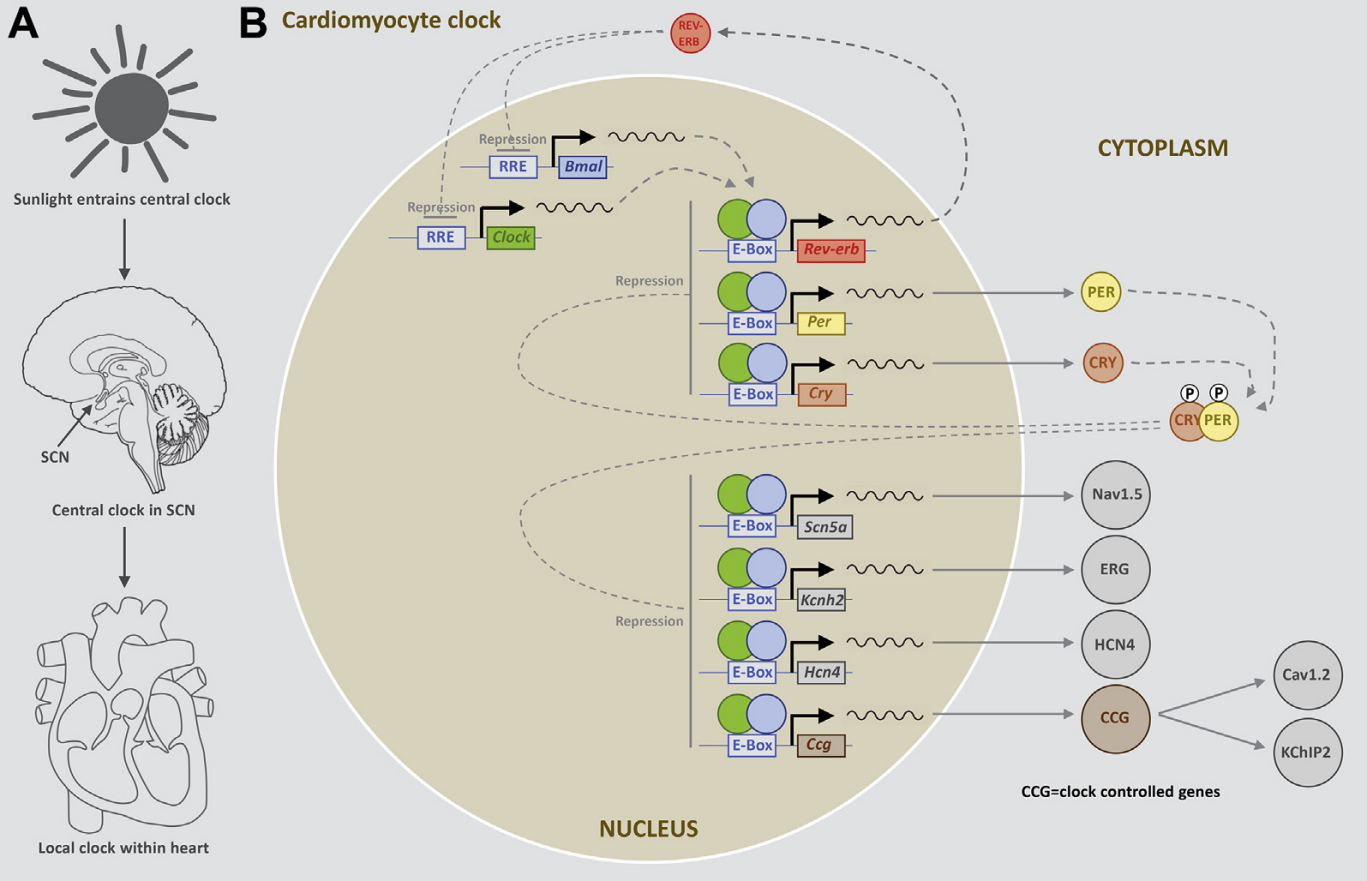
Schematic diagrams of the relationship between the environment, the central clock in the SCN, and the local clock in the heart **(A)** and the molecular pathways of the circadian clock **(B).** SCN = suprachiasmatic nucleus.

Circadian rhythms are controlled by circadian clocks, which drive day–night oscillations with a free running period of ∼24 hours. The molecular machinery of the circadian clock has been extensively characterized and reviewed elsewhere.^2^ At its core is a negative feedback loop^2^ involving 4 basic helix–loop–helix (bHLH) or per–arnt–sim (PAS) domain transcription factors: CLOCK, BMAL1, PER, and CRY3 (Figure 1). CLOCK and BMAL1 form a heterodimer within the nucleus and bind to canonical enhancer or E-box regions in the promoters of the *Per*, *Cry* and *Rev-erb* genes, thus activating their transcription (Figure 1). As a consequence, PER and CRY proteins gradually accumulate in the cytoplasm, and together they reenter the nucleus and suppress CLOCK/BMAL1-mediated transcription of the *Per* and *Cry* genes (Figure 1). At the same time, the accumulation of REV-ERB suppresses the transcription of *Clock* and *Bmal1*. Together, this establishes a negative feedback loop. This full cycle takes ∼24 hours.^2^, ^3^ Circadian clocks have been identified in the majority of mammalian cells. The suprachiasmatic nucleus (SCN) in the hypothalamus contains the central circadian clock and is synchronized with the environment by external cues such as light (Figure 1). In turn, the central clock entrains clocks within peripheral tissues via neurohumoral factors (autonomic tone, body temperature, and glucocorticoid signaling) (Figure 1).^4^ There is known to be a peripheral (or local) clock in the heart: oscillations in the expression of core circadian clock genes have been observed in the intact heart and are known to persist in isolated cultured myocardial tissue and cardiomyocytes.^5^, ^6^, ^7^ Up to 10% of the cardiac transcriptome is controlled by the heart’s local clock.^8^ As a result, key processes in the heart (including electrical excitability, signal transduction, and metabolism) vary in a circadian manner.^3^

## Circadian rhythm in the ECG including the heart rate

**Figure 2:**
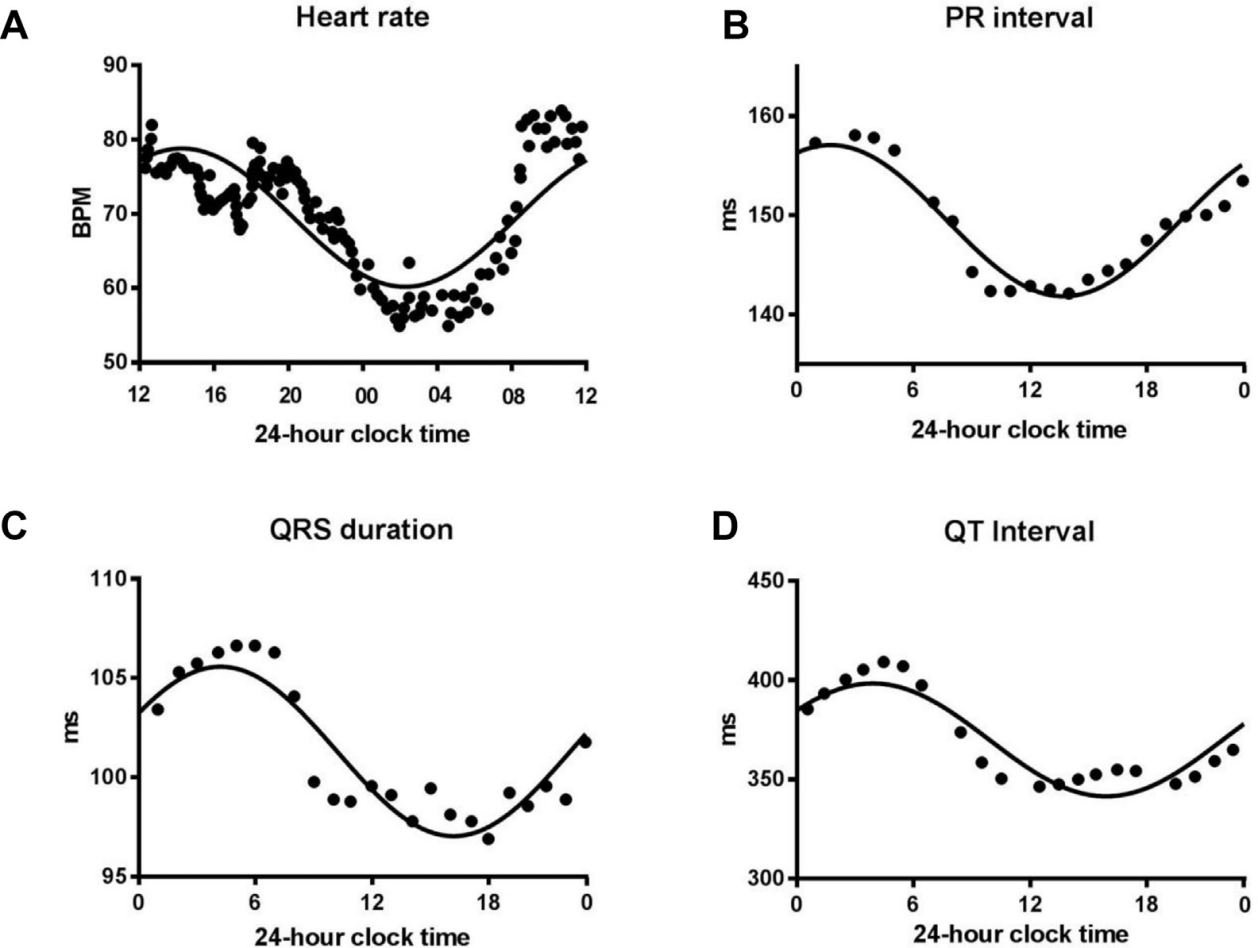
Circadian rhythm of ECG variables in the healthy human. **A:** Circadian rhythm of heart rate in 31 healthy men. BPM = beats/min. From Degaute et al,^9^ with permission. **B:** Circadian rhythm of PR interval in 50 healthy volunteers. From Dilaveris et al,^12^ with permission. **C:** Circadian rhythm in QRS duration in 20 healthy subjects. From Nakagawa et al^64^ with permission. **D:** Circadian rhythm of QT interval. From Bonnemeier et al,^11^ with permission.

Continuous 24-hour ECG recordings from healthy volunteers have shown a circadian rhythm in the ECG. The RR interval increases at night, corresponding to a slowing of the heart rate (Figure 2A).^9^ This nocturnal bradycardia seems to be independent of the nocturnal fall in blood pressure.^10^ At night, there is also a lengthening of the PR interval, QRS duration, and both uncorrected and corrected QT intervals (Figures 2B–2D).^11^, ^12^ This indicates slower atrioventricular (AV) node conduction, His–Purkinje conduction, and ventricular repolarization, respectively. A similar circadian rhythm in the ECG is seen in rodents.^13^ Hence, the normal electrical properties of the sinus node, AV node, His–Purkinje system, and ventricular muscle change over a 24-hour period.

## Circadian rhythm in sinus node pacemaking

### Normal pacemaking

The normal heartbeat is initiated at the pacemaker of the heart, the sinus node. Acute changes in heart rate occur through a change in *ionic conductance*. For example, stimulation of the vagus nerve causes an immediate decrease in heart rate. Vagal nerve endings release acetylcholine (ACh), which binds to M2 muscarinic receptors on the sinus node myocytes and hence activates ACh-activated K^+^ current (I_K,ACh_) and inhibits the funny current (I_f_) and the L-type Ca^2+^ current (I_Ca,L_).^14^ In contrast, stimulation of the sympathetic nerves to the heart causes an immediate increase in heart rate. Sympathetic nerve endings release noradrenaline, which binds to β-receptors on the sinus node myocytes to increase the I_f_ and I_Ca,L_, and induce changes in intracellular Ca^2+^ handling.^15^ Alternatively, chronic changes in heart rate can occur through a change in the *expression* level of membrane-bound ion channels or components of the intracellular Ca^2+^ handling apparatus. For example, there is evidence that the low resting heart rate of athletes is independent of the acute effects of the autonomic nervous system but rather is due to a downregulation in the expression of HCN4 (the main channel responsible for I_f_) in the sinus node myocytes.^16^ It is clear that a circadian rhythm in heart rate could occur through 1 of 2 mechanisms. Either the central circadian clock within the SCN drives a circadian rhythm in a neurohumoral factor (most likely autonomic tone), which in turn affects heart rate through ionic conductances, or the local cardiac clock may remodel ion channel expression within the sinus node over a 24-hour cycle.

### Role of local cardiac clock and ion channel remodeling

**Figure 3:**
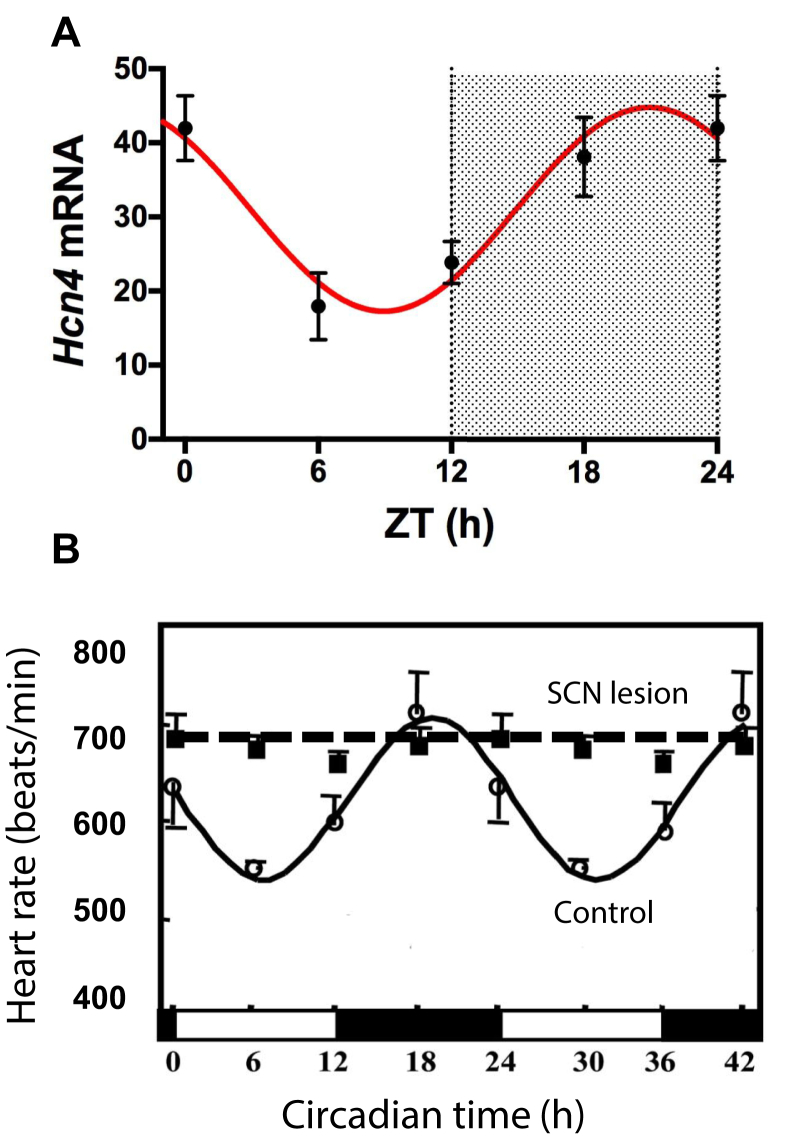
Contribution of local cardiac clock (via ion channel remodeling) and central SCN clock toward circadian rhythm in heart rate. **A:** Circadian rhythm in the expression of HCN4 mRNA (as determined by quantitative polymerase chain reaction) in the mouse sinus node at 4 time points over 24 hours (light and dark indicated by *shading*). Values given as mean ± SEM (n = 6–8). Data fitted with a sinusoidal curve. From D’Souza et al,^17^ with permission. **B:** Heart rate over 42 hours in control mice and mice in which the SCN has been lesioned (n = 3–6 mice). From Tong et al,^20^ with permission. SCN = suprachiasmatic nucleus; ZT = zeitgeber time.

In a recent preliminary report, we showed that in the mouse sinus node, the local cardiac circadian clock controls HCN4 mRNA, HCN4 protein, and the corresponding current, I_f_, in a circadian manner (Figure 3A).^17^ The density of I_f_ is higher in the awake period, appropriately explaining the higher heart rate.^17^ Two studies have reported that cardiomyocyte-specific disruption of the local cardiac clock (caused by the cardiomyocyte-specific knockout of one of the key clock genes, *Clock* or *Bmal1*) reduces but does not abolish the circadian rhythm in heart rate.^18^, ^19^ Hence, the local cardiac clock seems to contribute (but not fully explain) the circadian rhythm in heart rate.

### Role of the SCN

In contrast, the central SCN clock is essential for the circadian rhythm in heart rate. Mice subject to bilateral thermal ablation of the SCN lose the circadian rhythm in heart rate (Figure 3B).^20^ The same is true of mice deficient in vasoactive intestinal peptide, an important signaling molecule in the SCN.^21^ Furthermore, global knockout of the core circadian clock genes *Clock*^22^ and *Bmal1*^23^ (disrupting both the central SCN clock and local circadian clocks) results in dampening or loss of the circadian rhythm in heart rate.

### Role of the autonomic nervous system

It is commonly believed that the SCN clock controls the circadian rhythm in heart rate through a circadian variation in autonomic tone to the sinus node, in particular an increase in vagal tone at night. The SCN controls the circadian release of other neurohumoral factors, but the effect on the circadian rhythm in heart rate is not well understood (for further discussion, refer to the Supplemental Data). Apparent support for the role of autonomic tone comes from studies of day–night variations in heart rate variability (HRV), which is widely used as an indirect measure of cardiac autonomic tone.^24^ However, biophysical analysis of HRV has demonstrated an exponential-like relationship between HRV and heart rate, and changes in HRV observed in humans and rodents are mainly attributable to the accompanying changes in heart rate.^25^ Interestingly, direct recordings of stellate ganglion nerve activity and vagal nerve activity in an ambulatory dog model of heart failure show a circadian rhythm in sympathetic but not vagal tone to the heart.^26^ This is supported by measurements of skin sympathetic nerve activity in epileptic patients.^27^ These studies suggest that circadian sympathovagal balance may be largely due to diurnal fluctuations in sympathetic tone. Catecholamine secretion from the adrenal medulla also exhibits a prominent circadian rhythm, and this may contribute to the circadian rhythm in heart rate.^28^

**Figure 4:**
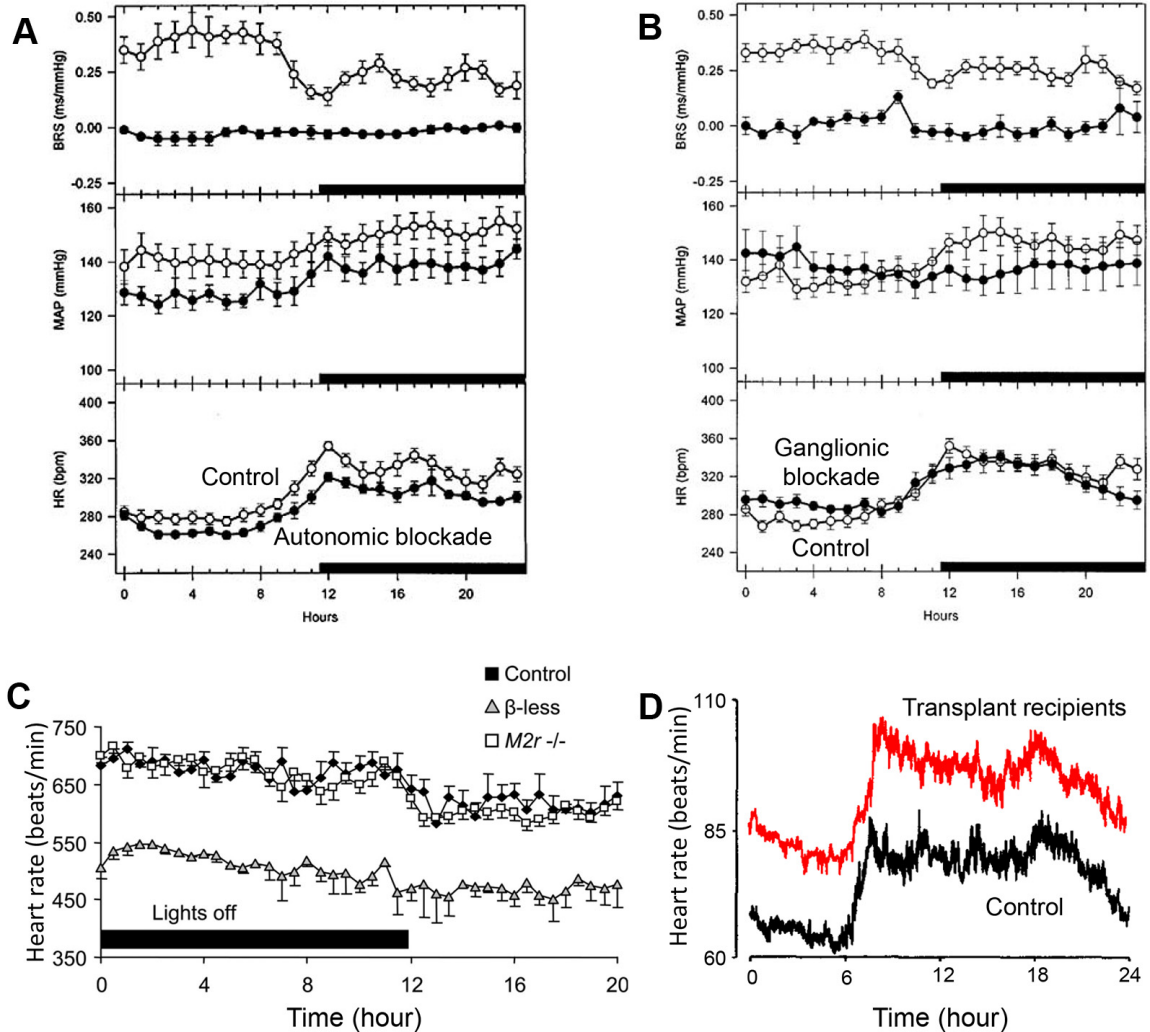
Evidence that the autonomic nervous system is not responsible for the circadian rhythm in heart rate. **A:** Complete autonomic blockade does not abolish the circadian rhythm in heart rate. BRS, MAP, and HR shown over 24 hours in spontaneously hypertensive rats during saline infusion (control) and during infusion of metoprolol and methyl-atropine (n = 9 rats per group). From Oosting et al,^29^ with permission. **B:** Pharmacologic blockade of the autonomic ganglia does not abolish the circadian rhythm in heart rate. BRS, MAP, and HR shown over 24 hours in spontaneously hypertensive rats during saline infusion (control) and during ganglionic blockade with hexamethonium (n = 8 rats per group). From Oosting et al,^29^ with permission. **C:** Circadian rhythm in heart rate is unaffected by knocking out the cardiac autonomic receptors. Heart rate is shown over 20 hours in control mice, mice deficient in cardiac sympathetic tone (lacking all 3 β-receptors), and mice deficient in cardiac vagal tone (lacking the M2 receptor) (∼8 mice per group). From Swoap et al,^32^ with permission. **D:** Cardiac transplant recipients still have a circadian rhythm in heart rate. Data from 17 heart transplant patients (11–36 months after transplantation) and 17 healthy volunteers matched for age and gender. From Idema et al,^33^ with permission. BRS = baroreceptor sensitivity; HR = heart rate; MAP = mean arterial pressure.

An alternative way to test the involvement of the autonomic nervous system is to block autonomic control of the heart. In spontaneously hypertensive rats, Oosting et al^29^ demonstrated that the circadian rhythm of heart rate is unaffected by pharmacologic block of the autonomic nervous system by constant venous infusion of methyl-atropine and metoprolol (Figure 4A), and pharmacologic block of the parasympathetic and sympathetic ganglia by constant venous infusion of hexamethonium (Figure 4B). In contrast, the circadian rhythm of mean arterial pressure (MAP) is abolished by ganglionic blockade (Figure 4B).^29^ They concluded that the circadian rhythm of heart rate is “largely independent from the autonomic nervous system.”^29^ Makino et al^30^ reported that autonomic blockade by subcutaneous injection of guanethidine and atropine diminished but did not abolish the circadian variation in heart rate in rats. Others have shown that, in the mouse, knockout of all 3 β-adrenergic receptors or the M2 receptor has little or no effect on the circadian rhythm in heart rate (Figure 4C).^31^, ^32^ Finally, cardiac transplant patients with autonomic denervation have a preserved nocturnal bradycardia 7–36 months after transplantation (Figure 4D).^33^, ^34^ These results challenge the commonly held notion that autonomic tone is the dominant mechanism by which the SCN drives circadian changes in heart rate.

**Figure 5:**
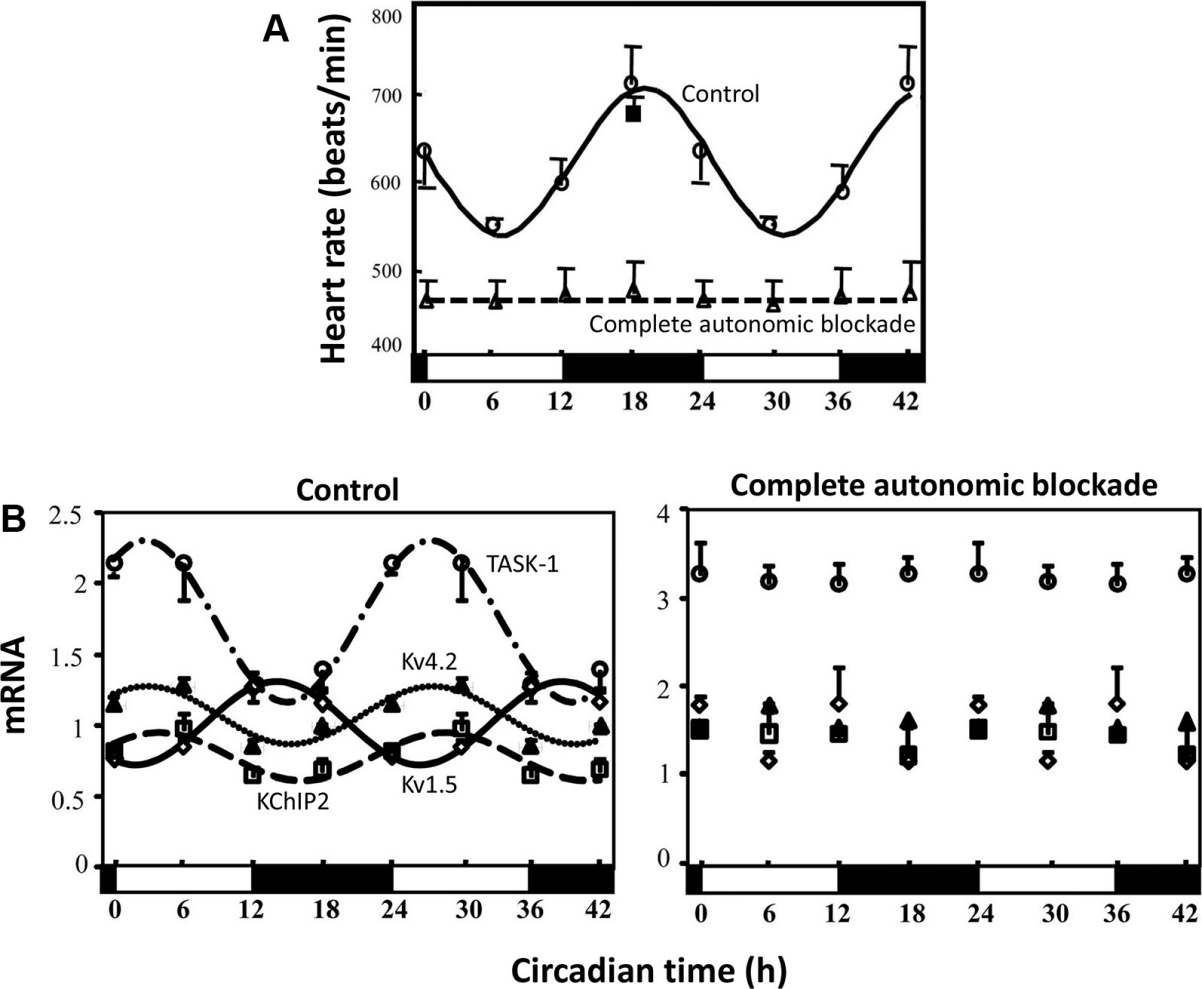
Unexpected role of the autonomic nervous system in mediating transcriptional effects in the heart. **A:** Complete autonomic blockade in the mouse by intraperitoneal injection of atropine and propranolol every 6 hours for 2 weeks abolishes the circadian rhythm in heart rate. Heart rate shown over 42 hours in control mice and mice with complete autonomic blockade (n = 3–6 mice). From Tong et al,^20^ with permission. **B:** Complete autonomic blockade in the mouse by intraperitoneal injection of atropine and propranolol every 6 hours for 2 weeks also abolishes the circadian rhythm in ventricular K^+^ channels. Expression of a range of K^+^ channel subunits shown over 42 hours in control mice and mice with complete autonomic blockade (n = 3–6 mice). From Tong et al,^20^ with permission.

However, there is evidence that the autonomic nervous system is involved in an unexpected manner. In the mouse, Tong et al^20^ reported complete loss of the circadian rhythm in heart rate under complete pharmacologic block of the autonomic nervous system by intraperitoneal injection of atropine and propranolol every 6 hours for 2 weeks (Figure 5A). This seems to contradict the conclusion reached from the result shown in Figure 4A. However, the loss of the circadian rhythm in heart rate in the experiment of Figure 5A may be unrelated to the autonomic nervous system’s well-known actions on ionic conductances, because in 2 studies Tong et al^20^, ^35^ showed that the circadian rhythm in ion channel expression is lost under the same conditions (Figure 5B). This suggests that the autonomic nervous system influences ion channel transcription. The difference between the contradictory results shown in Figures 5A and 4A may be related to the length of time the animals were subject to autonomic blockade; in Figure 5A the mice were subject to complete autonomic blockade for 2 weeks (clearly sufficient to exert an effect on gene transcription). Moreover, adrenergic agonists have been shown to stimulate the local cardiac clock and circadian gene expression. Isoproterenol amplifies circadian PER expression in mouse ventricular explants,^36^ and noradrenaline amplifies REV-ERB, PER, and BMAL1 circadian expression in rat cardiomyocytes.^6^ We hypothesize that the autonomic nervous system influences the circadian rhythm in heart rate by synchronizing the local cardiac clock to drive circadian oscillations in gene expression.

### Circadian rhythm of bradyarrhythmias

**Figure 6:**
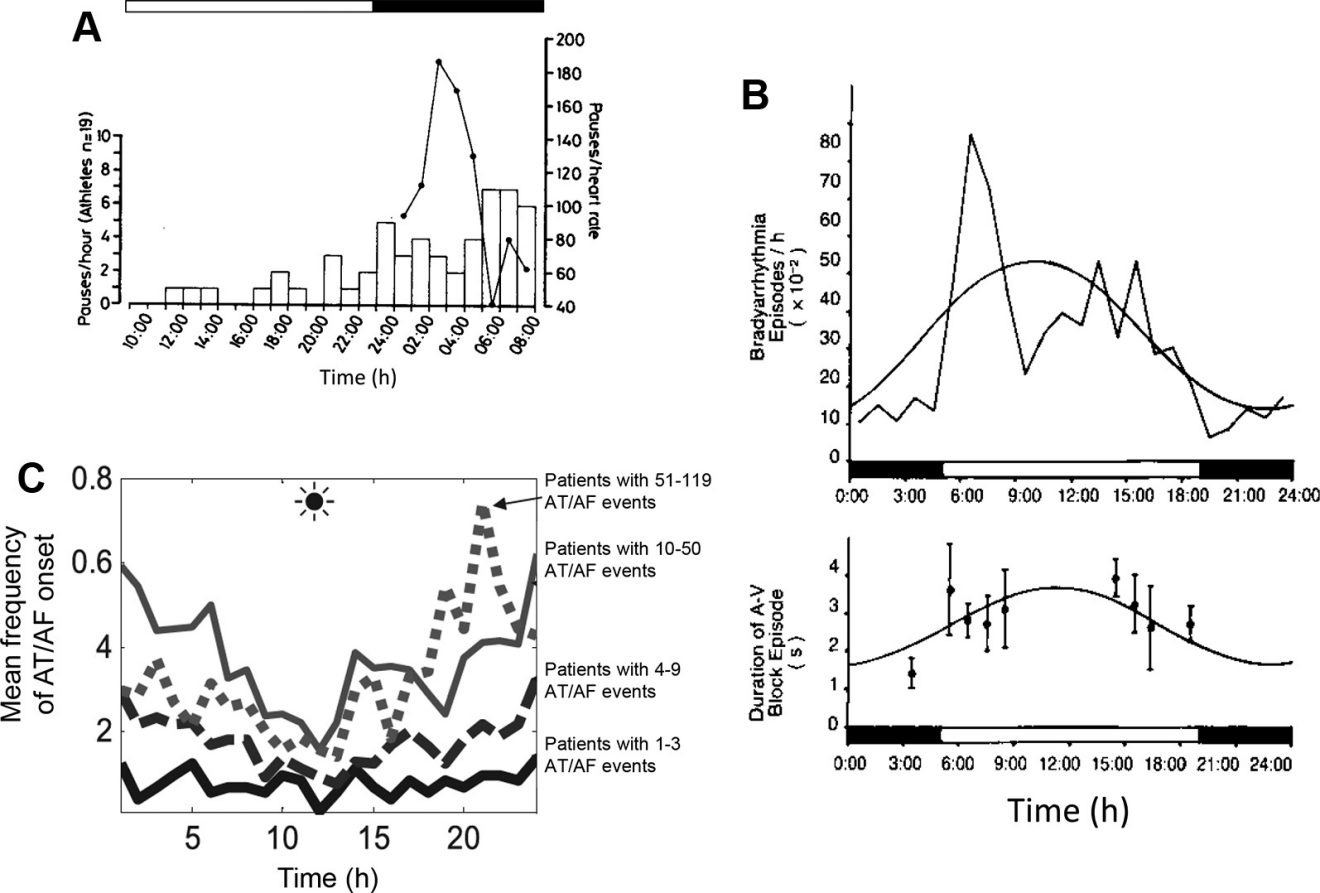
Circadian rhythm in bradyarrhythmias and atrial tachycardia/flutter and atrial fibrillation (AT/AF). **A:** Circadian variation in asystolic pauses throughout 24 hours in 19 veteran athletes (human). *Solid line* shows the distribution in a veteran athlete with 846 pauses in 24 hours, which only occurred between the hours of 01:00 and 08:00. From Northcote et al,^65^ with permission. **B:** Circadian variation of bradyarrhythmia episodes and duration of heart block episodes in the healthy rat (bradyarrhythmia episodes analyzed for 94 days in 14 rats). From Otsuka et al,^39^ with permission. **C:** Circadian rhythm of AT/AF onset stratified by frequency of AT/AF events. Data from 72 (1–3 AT/AF events per hour), 72 (4–9 AT/AF events per hour), 69 (10–50 AT/AF events per hour), and 7 (51–119 AT/AF events per h) patients. From Shusterman et al,^42^ with permission.

There is a clear circadian variation in bradyarrhythmias, with the majority occurring at night (Figure 6A).^37^, ^38^ In normal healthy adults 18–64 years of age undergoing continuous cardiac monitoring, the prevalence of nocturnal bradyarrhythmias is sinus bradycardia 16% (0% awake), sinus pauses >2 seconds 39.3% (0% awake), first-degree heart block 8.1% (2.4% awake), and Mobitz type 1 heart block 2.7% (0.3% awake).^37^ A similar circadian rhythm in bradyarrhythmias has been reported in the rat (Figure 6B).^39^ Given that bradyarrhythmias are so common in the general population, we would argue that in healthy people they occur as a direct result of the normal circadian rhythm in the electrical properties of the sinus and AV nodes. It is likely that the normal slowing of sinus node pacemaking at night (Figure 2A) is sufficient to cause sinus pauses and sinus bradycardia in some people, whereas the normal slowing of AV node conduction at night (Figure 2B) is sufficient to cause heart block in others. The central SCN clock is essential in driving this phenomenon because rats subject to bilateral thermal ablation of the SCN lose the nocturnal preponderance of bradyarrhythmias.^40^

Although unrelated to bradyarrhythmias *per se*, the slowing of AV node conduction at night could also explain the circadian rhythm of the ventricular response in atrial fibrillation (AF).^41^ The slowest ventricular response is reported to be at time 03:40 and the highest at 13:01, consistent with a lengthening of the PR interval and slowing of AV node conduction at night.^41^

### Circadian rhythm of supraventricular arrhythmias

A circadian rhythm is also seen in supraventricular arrhythmias. Paroxysmal AF, defined as AF terminating spontaneously within 7 days, occurs more commonly at night (Figure 6C). Shusterman et al^42^ analyzed 16,130 arrhythmic episodes from 236 patients with an implantable cardioverter-defibrillator (ICD) for AF, atrial tachycardia, and atrial flutter. It is the largest study in the field and uses data from ICD recordings, which removes the uncertainty of observational datasets using reported symptom onset. They found a clear increase in the incidence of arrhythmic events at night, regardless of whether or not underlying heart disease was present.^42^ Moreover, this nocturnal prevalence was most marked in those with the highest disease burden (Figure 6C).^42^ Two studies examined the circadian rhythm of paroxysmal supraventricular tachycardias separately from paroxysmal AF.^43^, ^44^ Both found that paroxysmal supraventricular tachycardias were more common in the afternoon and early evening, but both used the symptom onset as a surrogate for time of onset of arrhythmia.

The mechanisms underlying the circadian rhythm of supraventricular arrhythmias are poorly understood. Indeed, it is likely that the mechanisms may differ depending on the type of supraventricular arrhythmia. In the case of AF, an increase in vagal nerve activity (as supposedly occurs at night) acts as an arrhythmic trigger by increasing I_K,ACh_ in atrial cardiomyocytes, shortening atrial refractory period and promoting reentry.^45^ In addition, circadian ion channel remodeling may alter atrial arrhythmic substrate. Tong et al^20^, ^35^ have shown that in the mouse atrium, 4 K^+^ channel subunits (Kv1.5, Kv4.2, KChIP2, and TASK-1) and 2 connexins vary in expression in a circadian manner, and this is abolished by lesioning the SCN or blocking the autonomic nervous system. It is unknown how the SCN and autonomic nervous system cause circadian ion channel remodeling within the atria. We hypothesize that the mechanism is analogous to that in the sinus node discussed previously; the autonomic nervous system synchronizes the local cardiac clock to drive oscillations in gene expression. Hence, the circadian rhythm in autonomic tone may act as an arrhythmic trigger and alter the arrhythmic substrate.

## Circadian variation of ventricular premature complexes

**Figure 7:**
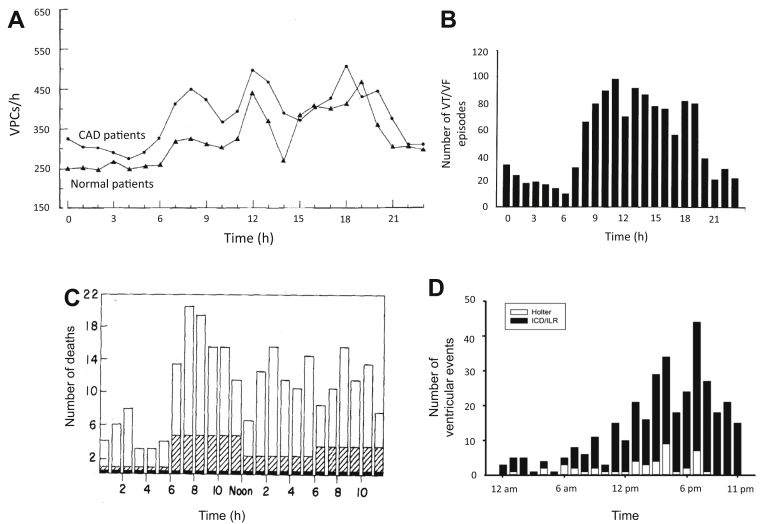
Circadian rhythm in VPCs, VT/VF, and sudden cardiac death. **A:** Circadian rhythm in the number of hourly VPCs in 38 patients during 2 days of Holter monitoring. From Lanza et al,^46^ with permission. **B:** Circadian rhythm of VT/VF time of onset. From Tofler et al,^51^ with permission. **C:** Circadian rhythm in time of death of definite or possible sudden cardiac death (n = 429). From Willich et al,^56^ with permission. **D:** Circadian rhythm in ventricular arrhythmia events in 80 catecholaminergic polymorphic ventricular tachycardia patients recorded using Holter monitoring, ICD, or ILR. From Miyake et al,^66^ with permission. CAD = coronary artery disease; ICD = implantable cardioverter–defibrillator; ILR = implantable loop recorder; VF = ventricular fibrillation; VPC = ventricular premature complex; VT = ventricular tachycardia.

Ventricular premature complexes show a robust circadian rhythm, occurring much more commonly during the day than at night (Figure 7A).^46^, ^47^ These results are based on 24-hour Holter monitoring of patients with frequent ventricular premature complexes (>30 per hour was typical) and a wide variety of heart diseases including myocardial infarction.^46^, ^47^

### Circadian variation in ventricular arrhythmias and SCD

Ventricular arrhythmias (ventricular tachycardia [VT] and ventricular fibrillation [VF]) and sudden cardiac death (SCD) demonstrate a prominent circadian rhythm, being more common in the morning on waking (Figure 7B). This is apparent from studies looking at the symptomatic onset of VT,^48^ VT onset during 24-hour Holter monitoring,^49^ and ICD shocks delivered in patients at risk for VT/VF.^50^, ^51^ Several of these studies found an additional late afternoon peak in VT onset.^52^, ^53^ One large study is notable for showing no circadian rhythm in VT/VF.^54^ Of the 811 patients allocated to the ICD treatment arm, 186 received 714 ICD shocks, and no morning peak in ICD shocks was present.^54^ This may have been due to use of modern heart failure therapy, in particular the widespread use of β-blockers. Presumably as a consequence of the circadian rhythm in VT/VF, a morning peak in SCD is seen in large epidemiologic studies (Figure 7C).^55^, ^56^ There is also a prominent circadian rhythm in ventricular arrhythmias associated with inherited channelopathies (Figure 7D; for further discussion, refer to the Supplemental Data).

A common explanation for the circadian rhythm in VT, VF, and SCD is the presence of a circadian rhythm in a proarrhythmic trigger, namely, a morning surge in sympathetic drive. β-adrenergic stimulation promotes Ca^2+^ overload, delayed afterdepolarizations, and reentry.^57^ Moreover, β-blockers have been shown to abolish the morning peak in SCD after myocardial infarction.^58^

There is also a circadian rhythm in the ventricular arrhythmic substrate. Studies have shown that ventricular action potential duration heterogeneity, as measured by its surrogate marker QT/QTc dispersion, increases in the morning.^59^ Moreover, there is a morning shortening of the ventricular refractory period that predisposes toward reentry.^60^, ^61^ Interestingly, the circadian rhythm in QT interval is abolished by lesioning the SCN or autonomic blockade,^20^ supporting the view that the central SCN clock and autonomic tone affects ventricular arrhythmic substrate. There is growing evidence that ion channel remodeling by the local cardiac clock may contribute. In animal models, transcripts for 1 Na^+^ channel, 1 Ca^2+^ channel, 5 K^+^ channels, and 2 connexins have been shown to have a circadian rhythm within the ventricles (Table 1 in Supplementary Data). In the case of KChIP2 (a regulatory β subunit of Kv4.2 responsible for the transient outward K^+^ current, I_to_), abolishing its circadian rhythm increases susceptibility to ventricular arrhythmias.^62^ At least some ion channel remodeling seems to be under the control of the local cardiac clock: CLOCK-BMAL1 has been shown to control transcription of both Nav1.5 and ERG, and, furthermore, cardiac-specific knockout of BMAL1 abolishes the circadian rhythm in both channels.^19^, ^63^

## Conclusion

**Figure 8:**
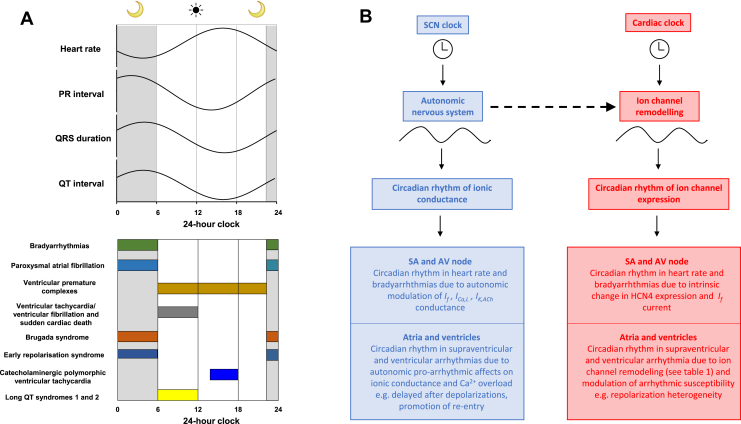
**A:** Schematic summary of circadian rhythm of ECG and arrhythmias in humans. The sleep period is illustrated as 23:00–05:59 and awake period as 06:00–22:59. **B:** Summary of mechanisms underlying circadian rhythm in heart rate and arrhythmias. *Dotted arrow* indicates possible actions of autonomic nervous system in synchronizing the local cardiac clock and mediating ion channel remodeling. AV = atrioventricular; SA = sinoatrial; SCN = suprachiasmatic nucleus.

Both normal cardiac electrophysiology and arrhythmogenesis demonstrate a robust circadian rhythm (summarized in Figure 8A). The SCN clock drives a circadian rhythm in neurohumoral factors, most importantly the autonomic nervous system, which alters ionic conductances and acts as a proarrhythmic trigger (Figure 8B, left). In addition, there is an emerging role for the local cardiac clock in driving circadian ion channel remodeling and altering arrhythmic substrate (Figure 8B, right). The autonomic nervous system may play a surprising role in acting as a bridge between the 2 clocks by synchronizing the local clock to drive oscillations in ion channel expression (Figure 8B, dotted arrow). However, the picture remains incomplete. Future work will need to identify a complete profile of the ion channels undergoing circadian remodeling within the heart. Moreover, the effects of circadian disruption (such as shift work and jet lag) on arrhythmogenesis remain poorly understood (for further discussion, see Supplementary Data). Finally, antiarrhythmic treatments targeting the circadian electrical properties of the heart remain a promising but unstudied therapeutic approach.
