# Supplementary material for: Circadian rhythm of cardiac electrophysiology, arrhythmogenesis, and the underlying mechanisms
Source: Heart Rhythm. 2019 Feb;16(2):298–307. doi: 10.1016/j.hrthm.2018.08.026 (PMC6520649; doi:10.1016/j.hrthm.2018.08.026)
Supplement: Supplemental Data [file mmc1.pdf]

Circadian rhythm of cardiac electrophysiology, arrhythmogenesis and the underlying mechanisms

Nicholas Black; Alicia D'Souza; Yanwen Wang; Hugh Piggins; Halina Dobrzynski; Gwilym Morris; Mark R. Boyett

DATA SUPPLEMENT

Role of other neurohumoral factors in driving the circadian rhythm in heart rate

The autonomic nervous system may not be the only way in which the SCN drives a circadian rhythm in heart rate. The SCN controls the circadian rhythm of many hormones including noradrenaline, adrenaline, cortisol and thyroid hormones.[1-3] However, the role of these hormones in mediating the circadian rhythm in heart rate is uncertain. For example, whilst a circadian rhythm in plasma catecholamines could explain the preserved circadian rhythm of heart rate in cardiac transplant recipients, it cannot explain this finding in autonomic blockade experiments (in which adrenoreceptors are blocked). In addition, whilst hyper- and hypothyroidism are well-known to affect heart rate, it is unknown whether the normal diurnal variation in the concentrations of thyroid hormones can drive a circadian rhythm in heart rate.[3] Further studies are required to clarify the role of these hormones on the circadian electrical properties of the heart.

Inherited channelopathies

A circadian rhythm has been described in several inherited channelopathies. Brugada syndrome is characterised by ST elevation in the right precordial leads and a predisposition towards VF and SCD.[4] VF in Brugada syndrome has the opposite circadian rhythm to that seen in ischaemic and non-ischaemic structural heart disease.[5] Studies using ICD recordings from patients with Brugada syndrome show that VF occurs more commonly at night.[4] ICD recordings have shown a similar nocturnal peak in appropriate shocks delivered in malignant early repolarization syndrome (ERS), a syndrome characterised by inferolateral early repolarisation on the ECG and 'short coupled' polymorphic VT and VF.[6] Both Brugada syndrome and ERS are worsened by periods of bradycardia and this may account for the circadian rhythm described. Catecholaminergic polymorphic ventricular tachycardia (CPVT) is a channelopathy in which arrhythmias are triggered by high adrenergic tone.[7]

We would therefore expect arrhythmias to occur most commonly during times of maximum daily activity. Miyake *et al*.[7] tested this hypothesis using 24 h Holter and ICD and implantable loop recorder (ILR) recordings from 80 children with CPVT. Maximum activity levels were observed in the early morning after waking (07:00-08:00) and mid-afternoon (15:00-16:00).[7] They recorded 423 VT events, which occurred more commonly in the afternoon (times 12:00-17:59) (Fig. 7D).[7] The same pattern was seen in children undergoing exercise stress testing.[7] The absence of a morning peak in arrhythmias in CPVT remains unexplained. Finally, both long QT syndromes 1 and 2 (LQTS1 and LQTS2) show a morning preponderance of symptomatic cardiac events, but no such diurnal variation is seen in LQTS3.[8]

Circadian disruption

Disrupted circadian rhythms are common in shift workers, international travellers and patients with sleep disorders, and there is growing evidence that this is associated with adverse health outcomes. For instance, sleep duration is a powerful predictor of all-cause mortality; a meta-analysis of 27 cohort studies including more than 70,000 elderly subjects has shown that sleeping less than 7 hours or more than 8 hours is associated with a 33% and 6% increased risk of death respectively[9].

Whilst many studies have shown that day-night dyssynchrony increases the risk of myocardial infarction, stroke, hypertension and dyslipidaemia,[10,11] surprisingly few studies have investigated the effects of circadian disruption on arrhythmogenesis. For instance, it remains unknown whether circadian disruption increases the risk of fatal arrhythmias or SCD. However, shift workers have been shown to have higher rates of VPCs[12,13] and longer QT_c intervals[14,15] compared to day workers. The mechanisms underlying this effect are likely to be complex and multifactorial. Shift work is associated with increased sympathetic tone (as measured by heart rate variability)[12] and causes diverse endocrine and metabolic effects, such as increased urinary catecholamine levels[12] and suppressed cortisol secretion,[16] which may affect the electrophysiological properties of the heart. We predict that circadian disruption is also likely to affect intrinsic ion channel remodelling within the heart and thereby increase arrhythmic susceptibility (although this remains unproven). Further epidemiological and mechanistic studies are needed to determine to what extent circadian disruption can precipitate arrhythmogenesis.

Table 1. Summary of ventricular ion channels with a circadian rhythm.

**Chanel type**	**Channel subunit**	**Gene**	**Current**	**Species**	**References**
**Na^+**	Nav1.5	*Scna5*	Na^+ current (I_{Na})	Rat, mouse	Schroder *et al.*[17]
**Ca^{2+}**	Cav1.2	*Cacna1c*	L-type Ca^{2+} current ($I_{Ca,L}$)	Guinea-pig	Chen *et al.*[18]
**K^+**	Kv4.2	*Kcnd2*	Transient outward K^+ current (I_{to})	Rat, mouse	Tong *et al.*[5]<br>Schroder et al.[19]<br>Yamashita *et al.*[20]
	KChIP2	*Kcnip2*	Regulator of transient outward K^+ current	Mouse	Tong *et al.*[5]<br>Jeyaraj *et al.*[21]
	Kv1.5	*Kcna5*	Ultra-rapidly activating delayed rectifier K^+ current ($I_{K,ur}$)	Rat, mouse	Tong *et al.*[5]<br>Yamashita *et al.*[20]
	ERG (Kv11.1)	*Kcnh2*	Rapidly activating delayed rectifier K^+ current ($I_{K,r}$)	Mouse	Schroder *et al.*[19]
	TASK-1 (K2p3.1)	*Kcnk3*	Outwardly rectifying K^+ current	Mouse	Tong *et al.*[5]
**Connexins**	Cx40	*Gja5*	Gap junction	Mouse	Tong *et al.*[22]
	Cx43	*Gja1*	Gap junction	Mouse	Tong *et al.*[22]

References

1. Scheer FAJL, Hu K, Evoniuk H, Kelly EE, Malhotra A, Hilton MF, Shea SA. Impact of the human circadian system, exercise, and their interaction on cardiovascular function. Proc Natl Acad Sci U S A 2010;107:20541-20546.

2. Chung S, Son GH, Kim K. Circadian rhythm of adrenal glucocorticoid: Its regulation and clinical implications. Biochim Biophys Acta 2011;1812:581-591.

3. Philippe J, Dibner C. Thyroid circadian timing: roles in physiology and thyroid malignancies. J Biol Rhythms 2014;30:76-83.

4. Matsuo K, Kurita T, Inagaki M, Kakishita M, Aihara N, Shimizu W, Taguchi A, Suyama K, Kamakura S, Shimomura K. The circadian pattern of the development of ventricular fibrillation in patients with Brugada syndrome. Eur Heart J 1999;20:465-470.

5. Tong M, Watanabe E, Yamamoto N, Nagahata-Ishiguro M, Maemura K, Takeda N, Nagai R, Ozaki Y. Circadian expressions of cardiac ion channel genes in mouse might be associated with the central clock in the SCN but not the peripheral clock in the heart. Biol Rhythm Res 2013;44:519-530.

6. Kim SH, Nam GB, Baek S, et al. Circadian and seasonal variations of ventricular tachyarrhythmias in patients with early repolarization syndrome and Brugada syndrome: analysis of patients with implantable cardioverter defibrillator. J Cardiovasc Electrophysiol 2012;23:757-763.

7. Miyake CY, Asaki SY, Webster G, et al. Circadian variation of ventricular arrhythmias in catecholaminergic polymorphic ventricular tachycardia. JACC Clin Electrophysiol 2017;3:1308-1117.

8. Takigawa M, Kawamura M, Noda T, et al. Seasonal and circadian distributions of cardiac events in genotyped patients with congenital long QT syndrome. Circ J 2012;76:2112-2118.

9. Silva AAd, Mello RGBd, Schaan CW, Fuchs FD, Redline S, Fuchs SC. Sleep duration and mortality in the elderly: a systematic review with meta-analysis. BMJ Open 2016;6:e008119.

10. Thomas K, Myriam J, Céline V, Till R. Shift-work research: Where do we stand, where should we go? Sleep Biol Rhythms 2010;8:95-105.

11. Vyas MV, Garg AX, Iansavichus AV, Costella J, Donner A, Laugsand LE, Janszky I, Mrkobrada M, Parraga G, Hackam DG. Shift work and vascular events: systematic review and meta-analysis. Br Med J 2012;345:e4800.

12. Rauchenzauner M, Ernst F, Hintringer F, Ulmer H, Ebenbichler CF, Kasseroler M-T, Joannidis M. Arrhythmias and increased neuro-endocrine stress response during physicians' night shifts: a randomized cross-over trial. Eur Heart J 2009;30:2606-2613.

13. van Amelsvoort LGPM, Schouten EG, Maan AC, Swenne CA, Kok FJ. Changes in frequency of premature complexes and heart rate variability related to shift work. Occup Environ Med 2001;58:678.

14. Meloni M, Setzu D, Del Rio A, Campagna M, Cocco P. QTc interval and electrocardiographic changes by type of shift work. Am J Ind Med 2013;56:1174-1179.

15. Murata K, Yano E, Shinozaki T. Cardiovascular Dysfunction Due to Shift Work. J Occup Environ Med 1999;41:748-753.

16. Bedrosian TA, Fonken LK, Nelson RJ. Endocrine Effects of Circadian Disruption. Annu Rev Physiol 2016;78:109-131.

17. Schroder EA, Lefta M, Zhang X, Bartos D, Feng HZ, Zhao Y, Patwardhan A, Jin JP, Esser KA, Delisle BP. The cardiomyocyte molecular clock, regulation of Scn5a, and arrhythmia susceptibility. Am J Physiol Cell Physiol 2013;304:C954-965.

18. Chen Y, Zhu D, Yuan J, Han Z, Wang Y, Qian Z, Hou X, Wu T, Zou J. CLOCK-BMAL1 regulate the cardiac L-type calcium channel subunit CACNA1C through PI3K-Akt signaling pathway. Can J Physiol Pharmacol 2016;94:1023-1032.

19. Schroder EA, Burgess DE, Zhang X, Lefta M, Smith JL, Patwardhan A, Bartos DC, Elayi CS, Esser KA, Delisle BP. The cardiomyocyte molecular clock regulates the circadian expression of Kcnh2 and contributes to ventricular repolarization. Heart Rhythm 2015;12:1306-1314.

20. Yamashita T, Sekiguchi A, Iwasaki YK, Sagara K, Iinuma H, Hatano S, Fu LT, Watanabe H. Circadian variation of cardiac K^+ channel gene expression. Circulation 2003;107:1917-1922.

21. Jeyaraj D, Haldar SM, Wan X, et al. Circadian rhythms govern cardiac repolarization and arrhythmogenesis. Nature 2012;483:96-99.

22. Tong M, Wang S, Pang Y, Zhou Y, Cui H, Ruan L, Su J, Chen X. Circadian expression of connexins in the mouse heart. Biol Rhythm Res 2016;47:631-639.
